# Building Bridges to Address the Osteoporosis Crisis

**DOI:** 10.1210/clinem/dgz307

**Published:** 2019-12-31

**Authors:** Ghada El-Hajj Fuleihan

**Affiliations:** Department of Internal Medicine, Division of Endocrinology, Calcium Metabolism and Osteoporosis Program, American University of Beirut, Beirut, Lebanon

**Keywords:** fractures, care gap, perceived risk, adherence, adverse events, reimbursement

“We are only as strong as we are united and as weak as we are divided”

J.K. Rowling

Osteoporosis disease burden by far exceeds that incurred by many non communicable diseases (NCDs), yet the care gap from this debilitating and deadly disease is staggering, and keeps widening (1, 2).

The American Society of Bone and Mineral Research-led coalition is the latest of a series of worthy initiatives. Importantly, it addresses several challenges raised above (7). The coalition made the critical decision to target elderly people above age 65 years, with the classical osteoporotic spine or hip fractures, a group where the evidence is unequivocal, benefits of therapy clearly outweigh risks, yet a high-risk group that remains largely untreated (1–3). The authors identified 13 clinical recommendations for secondary fracture prevention, 7 as fundamental (primary) (7). They strategically place patients, families, and care givers at the front and center. The first recommendation highlights the pressing need for education regarding consequences of these fractures, and measures to treat them. Other primary recommendations underscore the importance of communication with the usual healthcare provider at the time of fracture; regular fall risk assessment and management; the efficacy and timing, without any delays post fracture, for initiation of therapies, strategies to prevent or minimize their side effects; periodic re-evaluation for education, fracture, and fall risk assessment, and the monitoring of adverse events and adherence. Additional recommendations pertain to lifestyle measures such as muscle strengthening, balance, and posture exercises; education about risk–benefit ratio of therapies; and identification of a hierarchy for osteoporosis drug selection. It is however unclear why these were considered secondary. The authors also highlight the lack of data on optimal duration of long-term therapy, the need to consolidate treatment after cessation of denosumab and anabolic therapies, and referral of patients with major comorbidities and who fracture on therapy, to an endocrinologist or osteoporosis expert.

A few considerations not mentioned are worth noting. A single age-adjusted bone mineral density scan can predict fracture risk 25 years later. Serial height measurements in clinic, and lateral spine X-rays or vertebral fracture assessment at the time of dual-energy X-ray absorptiometry, increase the identification and use of pharmacologic therapy in patients with asymptomatic vertebral fractures. Observation of gait, balance, and timed up and go test refine the fall risk assessment and identify additional high-risk patients who may benefit from physiotherapy. Moreover, care of this complex disease can only evolve through a tight coupling to research addressing knowledge gaps. Defining periods of high-risk post-sentinel fracture, predictors of imminent fractures, on and off therapy, and predictors of Atypical Femoral Fracture (AFF) and Osteonecrosis of the Jaw (ONJ) are pressing needs. Identification of determinants of patient response to specific osteoporosis therapies, single or in combination, would refine recommendations from a 1 size fits all to a personalized approach. Implementation strategies are best derived from research taking into account peculiarities of local healthcare systems, tailored to primary, secondary, or tertiary care settings (8). Empowerment of patients, use of written materials, health apps, social media, applications of the health beliefs model, or goal setting strategies to improve adherence should be explored. Development and quality control of affordable generics is equally important.

Will the coalition succeed when others where met with limited success? Building bridges when obstacles are met, reaching consensus on recommendations, and a follow-up action plan that includes dissemination and implementation, are solid pillars the coalition plans to capitalize on. Highlights include expansion of secondary fracture registries, fracture liaison services, dissemination of educational materials, improvement in diagnosing vertebral fractures, development of quantifiable goals, identification of quality measures, potential use of new technologies, exploration of use of reimbursement and financial incentives. To build relationships with other key organizations is mentioned, but they are not specified. The American Nurses Association, the American Dental Association, and the American Association of Oral and Maxillofacial Surgeons, are relevant stakeholders. Endorsement and adoption by the various organizations represented in the coalition are also essential to successful implementation. Although the recommendations provide guidance to clinical situations in the United States, involvement of major international organizations in the coalition, namely the International Osteoporosis Foundation, Osteoporosis Canada, Osteoporosis Australia, are important to convey a consistent message worldwide. The World Health Organization is a major stakeholder for tackling NCDs in developing countries, where the care gap is even wider. It identifies health priorities adopted as national agendas by ministries of health in such countries. To date, osteoporosis is not on the World Health Organization NCD agenda. Most importantly, successful implementation also requires engagement of policy makers and payers. Tying the recommendations and implementation to accreditation and reimbursement are crucial.

The approach adopted by the American Society of Bone and Mineral Research with its assembled multidisciplinary coalition is indeed ambitious, as recognized by the authors, but most laudable. The clinical recommendations aim to prevent missed opportunities, optimize adherence through patient and physician education, and streamline the healthcare process through continuity in care. They constitute a solid step forward in a laborious and complex path. Much more is to be done, and we simply cannot fail. Success can only be achieved through a global and unified approach.

## Abbreviations

AFF: Atypical Femoral Fracture
FRAX: Fracture Risk Assessment Tool
NCDs: non communicable diseases
ONJ: Osteonecrosis of the Jaw

## Additional Information

***Disclosure Summary:*** The author has nothing to disclose

***Data availability:*** All data generated or analyzed during this study are included in this published article or in the data repositories listed in References.
